# Review Insights on Salivary Proteomics Biomarkers in Oral Cancer Detection and Diagnosis

**DOI:** 10.3390/molecules28135283

**Published:** 2023-07-07

**Authors:** Vidhya Rekha Umapathy, Prabhu Manickam Natarajan, Bhuminathan Swamikannu

**Affiliations:** 1 Department of Public Health Dentistry, Thai Moogambigai Dental College and Hospital, Dr. M.G.R. Educational and Research Institute, Chennai 600107, Tamil Nadu, India; 2 Department of Clinical Sciences, Centre of Medical and Bio-Allied Health Sciences and Research, Ajman University, Ajman P.O. Box 346, United Arab Emirates; 3 Department of Prosthodontics, Sree Balaji Dental College and Hospital, BIHER University, Pallikaranai, Chennai 600100, Tamil Nadu, India; bhumi.sbdch@gmail.com

**Keywords:** oral cancer, saliva, salivary proteomic, protein biomarkers, early detection

## Abstract

Early detection is crucial for the treatment and prognosis of oral cancer, a potentially lethal condition. Tumor markers are abnormal biological byproducts produced by malignant cells that may be found and analyzed in a variety of bodily fluids, including saliva. Early detection and appropriate treatment can increase cure rates to 80–90% and considerably improve quality of life by reducing the need for costly, incapacitating medicines. Salivary diagnostics has drawn the interest of many researchers and has been proven to be an effective tool for both medication monitoring and the diagnosis of several systemic diseases. Since researchers are now searching for biomarkers in saliva, an accessible bodily fluid, for noninvasive diagnosis of oral cancer, measuring tumor markers in saliva is an interesting alternative to blood testing for early identification, post-treatment monitoring, and monitoring high-risk lesions. New molecular markers for oral cancer detection, treatment, and prognosis have been found as a result of developments in the fields of molecular biology and salivary proteomics. The numerous salivary tumor biomarkers and how they relate to oral cancer and pre-cancer are covered in this article. We are optimistic that salivary protein biomarkers may one day be discovered for the clinical detection of oral cancer because of the rapid advancement of proteomic technology.

## 1. Introduction

For many years, saliva has been thought of as a potential replacement for blood serum and urine as a health monitor for the body. There are a number of reasons why saliva is a very desirable bodily fluid for disease diagnosis. Saliva collection is frequently affordable, safe, simple, and may be done at home (allowing for home-based sample); patients also typically view it as a painless and non-invasive practice [1]. It is a combination of salivary gland secretions, primarily from the three major glands (the submandibular, sublingual, and parotid). While desquamated epithelial cells, bacteria, their byproducts, food particles, and gingival crevicular fluid (GCF) are all significant elements of saliva [2]. In rare instances, saliva may also contain bronchial secretions, serum, and blood byproducts from oral injuries [3]. Compared to conventional biochemical analysis using tissue or blood samples, saliva analysis has a number of advantages, such as non-invasive and stress-free sample collection, simple storage and transportation, patient participation in supplying samples, cost effectiveness, and reduced risk of infection [4]. Since saliva contains a plethora of biological information, there is an increasing interest in it [5]. As a result, a new definition of “salivaomics” is proposed, which comprises salivary genomes, transcriptomics, proteomics, metabolomics, microbiomics, and microRNA (miRNA) [6].

Since the development of mass spectrometry and proteomics a decade ago, more than 3000 distinct proteins have already been found, signaling a significant advancement in the disclosure of the entire salivary proteome [7,8,9]. In the near future, a thorough analysis of the enormous quantity of data that has so far been collected will make it possible to customize therapeutic approaches by evaluating many parameters. Proteomic approaches are still very expensive, complicated, and difficult to access. However, with the development of straightforward, affordable, and more effective tools that can be used with small salivary samples for the early identification of many diseases. It is important to think about proteomics as a tool for a deeper comprehension of how protein structure modifies and interacts with other proteins, and eventually how that affects a person’s health. It must be viewed as more than just a systematic method of separating and cataloging all the proteins expressed in an organism. Since saliva is a fluid with diagnostic and prophetic value, qualitative and quantitative analysis of the saliva proteome is pertinent for both fundamental research and high-throughput [HTP] analysis [10,11,12].

**Figure 1:**
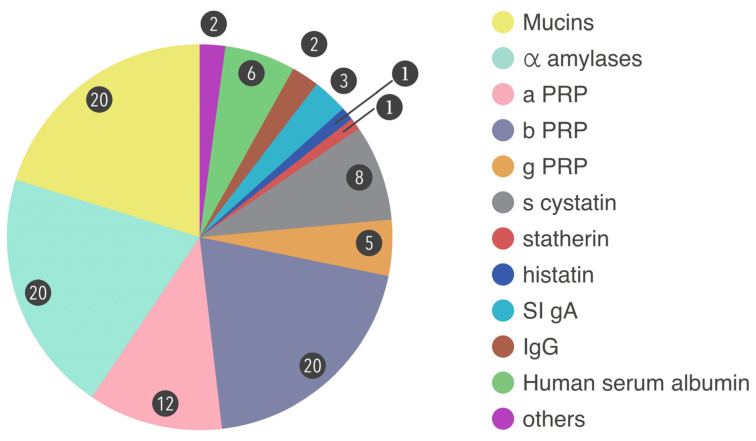
Major list of proteins found in human saliva. Numbers in the chart represent the % of proteins in saliva.

Salivary proteins form a complex network of connections. Post-translational modifications [PTM] equip proteins with complex structural and functional properties [13]. The largest salivary proteome dataset to date was reported by Bandhakavi, who identified 2340 proteins in saliva and used an analytical platform paired with a hexapeptide library for dynamic range compression and 3-D peptide separation [8]. About 30% of the proteins in blood are found in saliva; however, some salivary proteins with particular functions are solely created in the oral cavity [14]. There are six major families of proteins involved in salivary proteomics: (i) proline-rich proteins [PRPs] [includes acidic PRP[aPRP], basic PRP [bPRP], and glycosylated PRP[gPRP]], (ii) α-amylases, (iii) mucins, (iv) salivary [S-type] cystatins, (v) histatins, and (vi) statherin (Figure 1) [15,16]. The development of analytical HTP technology has significantly improved the detection sensitivity and specificity of proteomic biomarkers in saliva, even though some of their quantities are 10–15 times lower than protein concentrations in plasma [17,18,19]. The purpose of this review is to highlight the most demanding and complex features of the most recent proteome studies conducted on human saliva, with a focus on its potential use as a diagnostic fluid. The paper also offers a succinct summary of the key discoveries made possible by proteomics methods.

## 2. Oral Cancer and Salivary Markers

Over 300,000 people worldwide are affected by oral cancer [OC] each year, the majority of which is oral squamous cell carcinoma [OSCC], which may occur anywhere within the oral cavity [20,21]. Recurrence is prevalent in OSCC patients despite treatment, especially in those who have metastases to the neck lymph nodes, and symptoms commonly appear at a late stage. The overall 5-year survival rates for OC throughout the past several decades have remained low and essentially consistent [22,23]. Since delayed discovery is likely the main cause of the high morbidity rate among OC patients, reliable biomarkers are urgently required to improve early identification of OC.

At present, a comprehensive oral examination, typically performed by a dentist or other skilled health care practitioner, is the only reliable method for diagnosing OC [23,24]. A tiny tissue biopsy may be taken from the mouth cavity if an examination reveals an abnormal area so that a pathologist can examine it under a microscope for cancer cells. A succession of histopathologic stages, starting with benign hyperplasia and progressing through dysplasia, in situ carcinoma, and invasive squamous cell carcinoma, lead to oral carcinogenesis. At the molecular level, the growth of OSCC is an intricate process accompanied by genetic alterations and changes in the way several genes are expressed [25,26]. The variability of OSCC’s cells and molecules, as well as the sheer number of genes that may play a role in oral carcinogenesis, highlight how crucial it is to use proteomics to examine global alterations in gene expression. This implied that a successful approach to identifying or treating the condition needed to simultaneously target a number of proteins [pathways]. For the noninvasive detection of OC, researchers are looking for biomarkers in saliva, a bodily fluid that is simple to extract. For instance, the head and neck/OC frequently exhibit aberrant promoter hypermethylation and mitochondrial DNA mutations. Saliva testing for these genetic changes may help in disease diagnosis and surveillance [27,28,29,30]. Salivary proteins have been the subject of several studies looking into potential diagnostic indicators for OC [31,32,33,34].

The majority of OSCC had elevated levels of salivary soluble CD44, which was highly selective for differentiating cancer from benign disease [31]. Three tumor markers, including the cytokeratin 19 fragment Cyfra21-1, the cancer antigen 125, and the tissue polypeptide antigen, were reported to be considerably enhanced in the saliva of OSCC patients. The results were equivalent in terms of diagnostic value when these markers were measured in the sera of OSCC patients when they were aggregated [32]. The amount of p53 autoantibody in saliva was also shown to be connected with its serum levels in OSCC, suggesting that salivary analysis of the p53 antibody may offer a specific method for identifying a subgroup of OSCC with p53 problems [33]. These potential biomarkers have a limited ability to predict OSCC detection because they were only discovered one at a time.

## 3. Benefit of Salivary Biomarkers

The handling of saliva samples is secure. The human immunodeficiency virus is known to be inhibited by biomolecules in saliva. Therefore, compared to transmission from blood samples, the likelihood of transmission through saliva is quite low. Saliva samples are simpler to keep than blood samples since they do not clot [5]. Saliva collection without intrusive procedures is easy and painless for everyone. Patients’ discomfort and anxiety are significantly reduced as a result. Numerous proteins, mRNA and miRNA transcripts, and metabolites with a wide spectrum of biological roles are all present in cell-free saliva [34]. Salivary DNA is formed from a homogenous fraction of polymorphonucleated leukocytes and epithelial cells in the oral cavity. Since saliva is produced and collected locally from locations near oral cavity tumors, there is less interference when tumor-specific biomolecules are detected and analyzed. Analyte concentrations in saliva may be impacted by flow rates, age, drug usage, food, physiological status, and salivary gland pathology [35].

## 4. The Human Saliva Proteome

Analysis of the salivary proteomes of OSCC patients is a potentially viable method for discovering possible biomarkers for the disease because OC cells are submerged in the salivary environment. Compared to tissue biopsies, saliva is a fluid that is easily accessible. A huge number of saliva samples may be gathered and examined, which enables a strong study plan with enough statistical power to uncover the disease’s actual characteristics. In terms of weight, more than 90% of proteins found in saliva [approximately 3000] come from three pairs of “major” glands: the parotid, sub-mandibular, and sub-lingual glands [36]. About 200 proteins and peptides are made up of PRP protein parts and derivatives. The additional substances found in saliva make up the remaining 10% of its weight. These include lipocalin, which is secreted by small glands [the von Ebner glands of the lingual, buccal, palatine, and lingual regions] [36], while others, such as b-thymosins and -defensins, are mostly derived from GCF [37,38]. Some plasmatic proteins, like human serum albumin, are likely made from mucosal exudates, whereas others come from external sources, such as oral bacteria. [1,39,40]. List of significant protein biomarkers having therapeutic relevance that are present in human saliva is provided in the Table 1.

Distinguishing between diseased and normal circumstances is the most typical goal of proteome analysis. When there are numerous sources present, as there are with salivary glands, a quantitative change in one source may be offset by changes in others. Variable physiological circumstances have a different impact on the composition of the entire saliva. Minor glands secrete at a slow rate throughout the night on their own. Movements of the tongue and lips, along with mucosal dryness, increase production during the day and while at rest, especially by the submandibular gland [unstimulated secretion]. Strong stimuli cause parotid contributions to become more prominent, with a flow rate that is around twice as high as that produced when chewing by the submandibular gland. Saliva flow rates throughout the afternoon are generally higher than those during the daytime, with the highest coming in the midday. Another significant factor impacting the salivary proteome is age. Indeed, according to recent studies, the secretion of certain peptides is markedly different in children compared to adults [41,42,43].

For proteome analyses of human saliva, this dynamism presents a challenge, and all potential sources of variation must be carefully taken into account when selecting the best control group. However, since many PTMs that take place during glandular secretion are regulated by enzymes that are also found in other exocrine and endocrine glands, qualitative and quantitative changes may be a sign of concurrent malfunctions in other exocrine and endocrine glands and, consequently, a sign of systemic diseases.

## 5. Why Are Salivary Proteins the Target?

In contrast to DNA and RNA, proteins take part in biological functions in a variety of ways. Since they act as regulatory molecules in cellular pathways, proteins therefore serve a crucial role as the best biomarkers, particularly in certain instances. Key proteins found in bodily fluids can be analyzed to help diagnose diseases and play a vital role in assessing the health of biological entities. Proteomics may be more effective than traditional diagnostic techniques based on the subsequent identification of a number of clinically important biomarkers that are concurrently influenced by the disease. Due to salivary testing’s safety, affordability, and lack of invasiveness, it is becoming more and more accepted as a viable replacement for blood in diagnostic procedures [43,44,45,46]. It is crucial to analyze the human salivary proteome globally to comprehend disease pathogenesis. The protein composition of saliva has been examined both qualitatively and quantitatively using proteomic technologies. To realize the full diagnostic potential of complete human saliva, comprehensive protein identification is essential. In order to solve this issue, an analytical platform that combined three-dimensional peptide fractionation with hexapeptide libraries found 2340 proteins in WS, making it the biggest saliva proteome dataset [8]. Such findings upsurge saliva’s utility in health care and diagnostics and also offer a broad framework for enhancing proteome coverage of intricate biological materials.

The quantifiable, non-invasive analysis of readily available human body fluids is this platform’s key strength. Diseases such as dental caries, periodontitis, bleeding oral cavity, oral lichen planus, Sjögren’s syndrome, squamous cell carcinoma, breast cancer, gastric cancer, diabetes, and graft-versus-host disease have all seen success with saliva proteomic analysis as a new approach to finding markers of interest [47]. Proteomic analysis of human WS has demonstrated that salivary biomarkers may aid in disease diagnosis, provided that appropriate collection techniques are standardized [41]. A detailed investigation of the salivary proteome is necessary to comprehend the roles of salivary proteins and find disease biomarkers involved in various pathophysiological states, which will eventually enhance patient diagnosis and prognosis. When used with easily available saliva samples from disease patients for biomarker analysis, such a global approach makes it possible to get the most discriminating protein biomarkers that can best predict the disease status [48,49]. These initiatives aim to find proteins that are specifically linked to a particular human disease in order to correctly diagnose and treat the ailment. The WS proteome is extremely vulnerable to numerous physiological and pharmacological events. To obtain insight into the physiological and pathological processes pertinent to health and to identify significant biomarkers for the disease, it will be essential to understand the proteome of the entire saliva [50]. Saliva is currently acknowledged as an ideal diagnostic material that may be collected easily and noninvasively because of technological breakthroughs in biomarker research [51,52,53,54]. The use of saliva to track disease development has been a highly desirable goal in healthcare promotion. Since the advantages of saliva proteomics as a diagnostic tool have been made clear. The complete proteome of human salivary fluid, which is accurate and readily available, has the potential to lead to new avenues for the identification of disease biomarkers and for diagnosis. It also offers information that will be helpful for future research. Saliva proteomics is a prospective biofocus that will drive salivary studies and provide significant long-term advantages for public health.

Accordingly, several biomarkers—such as tissue polypeptide antigen [TPA], cytokeratin-19 fragment [Cyfra21e1], and cancer antigen 125 [CA 125]—were four times more prevalent in OC patients [32]. The protein salivary biomarkers can be characterized separately or together to assist in the early identification of OSCC. When Hu et al. examined the human salivary proteome, they discovered that OSCC patients had variable levels of different salivary proteins, including Mac-2 binding protein, CD59, profilin 1, myeloid-related protein 14, and catalase [55]. Another study found that most OSCC patients had elevated levels of soluble CD44, which helped to distinguish cancer from benign conditions [56]. The potential for cytokines [IL-6 and IL-8] to serve as useful biomarkers for the OSCC was examined by John et al. in 2004. Increased IL-6 has been linked to the encouragement of immunological nonresponse, which in turn induces cachexia and hypercalcemia. OSCC patients with a poor prognosis have been observed with these symptoms. The proliferation of angiogenesis as well as the chemotaxis of macrophages and granulocytes, which are clearly seen in the stroma of OSCCs, depend on IL-8. Several oral diseases may cause an increase in salivary cytokine levels. The results of this study were significant for IL-8 compared to IL-6, indicating that the involvement of underlying diseases is enhanced by the rise of the IL-8 marker in the saliva of OSCC. In addition to their potential clinical uses, these proteins may aid in our understanding of the molecular basis of numerous disorders [57].

## 6. Salivary Proteomics Signature Markers

Salivary gland acinar cells produce and secrete more than 2000 proteins, which are crucial for preserving the oral cavity’s homeostasis [58,59]. Through the use of cutting-edge proteomic methods such as liquid chromatography, mass spectrometry, and protein/peptide labeling, which enable the detection of low-abundance molecules in the salivary proteome, the salivary proteome has proved effective for discovering biomarkers for OSCC. The saliva of OSCC patients and healthy controls has varied proteome profiles, according to numerous studies [34]. Saliva from OSCC patients has been found to include a number of protein biomarkers. In patients with oral lichen planus, endothelin-1 has been identified as a possible biomarker for OSCC development [60]. Salivary biomarkers of OC have been discovered to include IL-8, IL-1, glycoprotein M2BP, CD59, myeloid-related protein 14 [MRP14], and catalase [61,62]. According to Shiptzer et al., the saliva of OC patients contained higher amounts of Cyclin D1, Ki67, LDH, and MMP-9, whereas lower levels of 8-oxoquanine DNA glycosylase [OGG1] and Maspin [63]. Extracellular matrix and basement membranes are degraded by MMP2 and MMP9. VEGF is released as a result, promoting angiogenesis. Tumor invasion and metastasis are linked to MMPs [64].

For chair-side/point-of-care oral fluid (gingival crevicular fluid, mouthwash, and saliva) diagnostics, matrix metalloproteinase-8 is a promising candidate [65]. It can be used to predict, diagnose, and track the progression of episodic periodontitis and peri-implantitis, as well as to keep track of treatments and medications. Salivary matrix metalloproteinase-8, in conjunction with interleukin-1beta and P. gingivalis, can be a helpful diagnostic tool, particularly in large-scale public health surveys when a comprehensive periodontal examination is not practical.

The potential benefit of using active matrix metalloproteinase-8 (aMMP-8) as a biomarker in the new periodontitis categorization system was examined by Sorsa et al. [66]. When compared to individuals with more advanced stages and grades of periodontitis, healthy participants’ mouthrinse aMMP-8 levels were much lower. Furthermore, compared to bleeding on probing (BOP), aMMP-8 levels had a lower correlation with plaque levels. In comparison to classic periodontal parameter bleeding on probing, aMMP-8 was therefore more resistant to the confounding effects of mouth hygiene. In under 5 min, the aMMP-8 point-of-care mouthrinse test may be used as an adjuvant and preventative diagnostic tool to identify continuing periodontal breakdown and periodontal disease, both of which are categorized by stage and grade.

According to Brailo et al. [67], patients with oral leukoplakia had considerably higher levels of IL-6 and TNF-α in their saliva. The tumor suppressor gene p53 is rendered inactive by IL-6 by hypermethylating its promoter, which leads to unchecked cell proliferation. Through transcriptional activation of NF-kB, TNF-α promotes cell proliferation, prevents apoptosis, and increases the secretion of proinflammatory cytokines. According to Rhodus et al. [68], patients with OC had considerably higher levels of IL-1, IL-6, IL-8, and TNF- α in their saliva than those with oral dysplasia. In comparison to controls, individuals with tongue squamous cell carcinoma had significantly increased levels of these cytokines and also VEGF-A in their saliva [69]. It is well recognized that cytokines play a role in both angiogenesis and inflammation.

Telomerase was found to be overexpressed in the saliva of OC patients, according to Zhong et al. [70]. Actin and myosin levels in the saliva of patients with OC were consistently higher than those in individuals with premalignant lesions, according to the De Jong et al. study [51]. Myosin and actin promote cell invasion and motility. As an early detection biomarker for OC, salivary transferrin has also been described. Growing tumor growth is connected with an increase in salivary transferrin. According to Jou et al. [71], transferrin affects the survival and growth of cancer cells. The saliva of OSCC patients significantly overexpressed cancer antigen CA-125, TPA, and tumor marker Cyfra 21-1 [6]. When compared to healthy individuals, the saliva of OSCC patients had considerably higher amounts of basic fibroblast growth factor [bFGF]. Those with newly diagnosed OSCC have been found to have much higher levels of bFGF in their saliva than patients with the disease in remission or who have oral leucoplakia. A possible biomarker for post-treatment OSCC patients looking to detect recurrence is salivary bFGF [72,73].

In addition to the biomarkers mentioned above that were found by HTP techniques, other possible proteomic biomarkers have recently been suggested by conventional techniques. Resistin [RETN], also known as adipose tissue-specific secretory factor, is a cysteine-rich adipose-derived peptide hormone [ADSF]. It was first proven to act as an endocrine hormone, and further research linked it to type II diabetes, inflammation, and heart disease. More and more evidence showed a strong correlation between RETN, lymph node metastases, and late-stage OSCC. This finding indicated that RETN may serve as a salivary biomarker for the early diagnosis of OSCC [74]. Salivary albumin, a component of the compensatory antioxidant defense system, was linked to a large rise in oral precancer and OC instances. Reactive oxygen species [ROS] have also been proven to be related to the genesis and promotion of OC. This finding suggested that albumin, because of its antioxidation function in reducing oxidative stress, may be crucial in the diagnosis and prognosis of premalignant and malignant oral disease [75]. The early diagnosis of OC has drawn a lot of interest in immunogenic proteins. Four potential proteins, including keratin-10 [K-10] and human serum albumin, as well as the human pancreatic alpha-amylase [HPA] and salivary amylase, have recently been suggested as salivary biomarkers for OSCC, although more validation is necessary [76]. These findings showed that OSCC patients’ salivary proteins have notable characteristics.

## 7. Challenges in Salivary Proteomic Research

Despite the immense potential of salivary proteome analysis, there are still certain obstacles to its transition from the lab to the clinic [77]. One of the most problematic obstacles is the complicity of the proteome brought on by PTMs. PTMs primarily comprise the production of disulfide bonds, proteolytic cleavages, glycosylation, phosphorylation, and sulphation. Many of these PTMs are also extremely reversible and dynamic, which supports the activity, stability, and location of complex proteins [78]. Therefore, there is still much to learn about post-translationally changed proteins. However, ongoing technical progress has made it easier to conduct PTM research. By using MS/MS investigations, the Phospho Site Plus [PSP] public database has described over 240,000 phosphorylation and 22,000 ubiquitination sites for more than 20,000 proteins [79]. Additionally, dynamic range compression prior to hexapeptide libraries can improve the detection sensitivity of PTMs and proteins with low abundances. Nearly twice as many salivary phosphoprotein and N-glycoprotein identifications were seen when MS, dynamic range compression, and hexapeptide libraries were combined. With a focus on phosphorylation and glycosylation, sample enrichment and network-based protein-protein interactions have also been shown to be effective methods for discovering the entire spectrum of PTMs [80].

## 8. Global Saliva Proteome Analysis

In terms of biochemistry, saliva’s most significant components are proteins. Numerous substances found in human saliva can be useful for tracking general health and wellbeing, disease aetiology, and dental health. The initial step in finding unique molecules in saliva connected to human health and diseases is a thorough investigation and identification of the proteome content in human saliva. The goal of proteomic research on human saliva is to find and characterize novel peptides and proteins that exhibit biological activity at the glandular level or under different clinical circumstances. The first database in the world to centralize proteomic data, annotate discovered saliva proteins, and provide public access is the saliva proteome knowledge base [http://www.skb.ucla.edu] (accessed on 1 July 2023). Researchers frequently use a focused method, where a small number of proteins are further confirmed in a clinical environment, as well as a discovery approach, which entails unraveling the entire proteome, when attempting to identify disease-associated proteins. Every strategy has benefits and drawbacks of its own. Hardt et al. [81] employed 2D SDS-PAGE to separate the proteins in parotid glandular saliva before MS analysis in order to identify the proteins therein. These techniques have been used to identify proteins with moderate to high molecular weight as well as peptides in the 1–6 kDa range [histatins, cystatins, and PRPs] [82].

Scientists have discovered more than 1050 proteins in WS using LC-MS and 2D-MS [48,49]. For the separation and identification of proteins and peptides with low relative molecular masses, this combination is especially useful [83]. The salivary peptidome and proteome have been extensively qualitatively and quantitatively characterized in a variety of normal and pathological states during the past few years by researchers using proteomic techniques. PRPs, statherins, cystatins, and histatins are the four main types of salivary proteins that have been identified by researchers performing proteomic investigations on human saliva [84,85]. Saliva from individual glands as well as human WS have both been subjected to extensive global analysis [48,49,86], revealing protein patterns specific to each gland type [81,87].

Many of the early proteomic studies centered on WS. Approximately 1100 proteins were found in WS by Schipper et al. [82] and 1166 by Denny et al. [87]. There are currently 2290 proteins listed in the WS, and human saliva contains about 27% of the plasma proteins, according to recent research by numerous laboratories. Due to the many methods and technologies used in the discovery of these biomolecules, there is little overlap between the known proteins in parotid glands when they are compared.

## 9. Future Perspective and Conclusions

Saliva has tremendous potential for monitoring general health and disease and offers unmatched chances for clinical applications. It has the benefits of being simple, safe, cost-effective, and non-invasive. This review provides knowledge on the state of saliva diagnostics and explores how they might be used to find particular HTP noninvasive biomarkers for disease detection and treatment purposes. It is once again emphasized how important current developments in salivary proteome profile analysis and their prospective applications are. The thorough examination and characterization of the proteome composition of human saliva may aid in the comprehension of pathophysiology and lay the groundwork for the identification of possible biomarkers of human disease. Additionally, by adding proteomic findings to salivary diagnostics, we will be able to link molecular analytes to medications, therapeutic results, and ultimately disease progression. Further advancements in this field have the potential to fundamentally alter how we screen for, evaluate the risk of, and treat a variety of medical problems. This strategy should make it possible to offer more tailored care before reaching an advanced stage. We anticipate that salivary diagnostics will soon be refined to the point where a smaller panel of diagnostic biomarkers can be evaluated and results obtained quickly, significantly enhancing the quality of human life.

## Figures and Tables

**Table 1:** List of major protein biomarkers found in human saliva with clinical significance.

Proteins	Functional Role and Significance in Cancer
Interleukins (IL-6, IL8, IL-1a, IL-1b)	These cytokines, which have been proven to be markers of the carcinogenic transition of oral precancerous lesions into OC, are proinflammatory and proangiogenic in nature.
Tumor Necrosis Factor-Alpha	
Fibroblast Growth Factor 2 (Basic)	Endothelial cell proliferation, invasion, and angiogenesis progression
Tissue polypeptide antigen (TPA)	Cyfra 21-1, CA 125, and TPA markers are used as diagnostic tool, telomerase activity is detected in tumor cells, and telomerase activity is responsible for maintaining telomere length throughout chromosomal replication.
Cyfra 21-1	
Cancer antigen 125 (CA 125)	
Telomerase	
S100 Calcium Binding Protein A9	Recruitment, adhesion, and migration of leukocytes. Induction of cytokine and chemokine secretion.
Cyclin D1	Cell proliferation and metastasis.
Marker Of Proliferation Ki-67	Cell proliferation and metastasis.
Lactate Dehydrogenase	Production of lactate from pyruvate under anaerobic conditions which is key feature of cancer cells.
CD44	In contrast to MRP14, a calcium-binding protein with a sensitivity of 90% and specificity of 83% in cancer detection, CD44 and CD59 distinguish benign diseases from cancer with extremely high sensitivity and specificity.
CD59	
Profilin	
MRP14	
Matrix Metallopeptidase 2	Regulation of vascularization and metastasis
Matrix Metallopeptidase 9	Migration, chronic inflammation, angiogenesis, and metastasis.
8-Oxoguanine DNA Glycosylase	Repair of oxidative DNA damage
Maspin or Serpin Family B Member 5	Inhibition of tumor angiogenesis
Endothelin-1	Promote tumorigenesis
Glutathione	Epidemiological marker for chemoprevention identifies the risk of development of OSCC.
Vascular Endothelial Growth Factor A	Angiogenesis, endothelial cell proliferation, and migration.
Mac-2 binding protein (M2BP)	M2BP is used to detect OSCC. The sensitivity and specificity of this biomarker are 90% and 83%, respectively, and they all function as clinical tools for the noninvasive diagnosis of OSCC.
Squamous cell carcinoma antigen 2	
Involucrin	
Calcyclin	
Cathepsin-G	
Azurocidin	
Transaldolase	
Carbonic anhydrase I	
Calgizzarin	
Myeloblastin	
Vitamin D-binding protein	

## Data Availability

The data presented in this study are available in this article.

## Sample Availability

Samples of the compounds are not available from the authors.
